# Validation and Psychometric Evaluation of the Polish Language Version of the Sleep Disturbance Scale for Children (SDSC)

**DOI:** 10.3390/jcm15010387

**Published:** 2026-01-05

**Authors:** Małgorzata Jączak-Goździak, Oliviero Bruni, Marcin Żarowski

**Affiliations:** 1 Department of Developmental Neurology, Poznan University of Medical Sciences, 61-701 Poznan, Poland; 2 Department of Developmental and Social Psychology, Sapienza University of Rome, 00-185 Rome, Italy; oliviero.bruni@uniroma1.it

**Keywords:** sleep disorders, SDSC, Polish validation of SDSC

## Abstract

**Background/Objectives**: In this study, we aimed to validate and psychometrically evaluate a tool for examining sleep disorders in Polish children. **Methods**: This study involved a randomly selected sample of 348 children aged 6 to 15 years, sourced from preschools, primary schools, and secondary schools in a city with a population exceeding 100,000, in addition to two smaller towns in Poland. Parents were asked to complete the Sleep Disorders Scale for Children (SDSC) in conjunction with a sociodemographic survey. The tool’s reliability was assessed using Cronbach’s alpha (α), and correlations among various domains were evaluated using Spearman’s rank correlation coefficient (Rs). **Results**: The study demonstrated excellent internal consistency for the SDSC, with a Cronbach’s α value of 0.9. The individual subscales also exhibited acceptable reliability values, ranging from 0.69 to 0.83. Considering T-scores over 70 as indicative of a problem, we identified at least one sleep disorder in 65 participants (18.68%). The most common issues included sleep hyperhidrosis (SHY; 7.47%), disorders of excessive somnolence (DOES; 7.18%), and sleep–wake transition disorders (SWTDs; 5.75%). Students in secondary education were more likely to experience disorders of initiating and maintaining sleep (DIMS), disorders of arousal (DA), and DOES. **Conclusions**: Based on our findings, the Polish version of the SDSC may be considered a reliable and effective tool for assessing sleep disturbances in school-age children and adolescents.

## 1. Introduction

Healthy sleep, both in duration and in quality, is vital for mental and physical well-being and plays a crucial role in proper development [1,2,3]. The rise in technology and the increasing use of digital devices—such as computers, smartphones, and televisions—by younger children, combined with the widespread use of social media among adolescents, has contributed to a noticeable increase in sleep disorders within these age groups [3,4,5]. Excessive screen time is a key factor leading to insufficient sleep, poorer sleep quality, heightened daytime sleepiness, and disruptions in physiological sleep and circadian rhythms [3,4,5,6]. These factors may result in impaired melatonin secretion, delayed sleep onset, and reduced REM sleep, ultimately causing serious consequences such as cognitive impairments, mood disorders, and metabolic issues [4]. It is essential to ensure adequate sleep for children and adolescents and identify any abnormalities. Currently, in Poland, there is no available tool for assessing or screening sleep disorders in children and adolescents, thus highlighting an important gap in this area.

When assessing sleep-related problems, it is recommended to use sleep rating scales and sleep diaries for the initial assessment before employing objective methods or tests, such as polysomnography or actigraphy, which are more time-consuming [7]. Numerous questionnaire-based tools are available in the literature to assess sleep quality and related problems [8]. These tools have been used in screening tests for a number of years, providing reliable and standardized measurements at an inexpensive and accurate level [8]. Among the various screening survey tools, we can differentiate between the following types [9]:

1. Questionnaires based on parent reports [9]: Children’s Sleep Habits Questionnaire (CSHQ) [10] and the Sleep Disturbance Scale for Children (SDSC) [11].
2. Self-reporting questionnaires for patients [9]: Children’s Sleep Comic (CSC) [12], the Sleep Self-Report Scale (SSRS) [13], the Epworth Sleepiness Scale for Children (ESS-C) [9], and the Nightmares Effects Questionnaire (NEQ) [9].

## 2. Materials and Methods

The Sleep Disturbance Scale for Children was selected for validation, as it is one of the few that meets all methodological psychometric criteria [14] and has high internal consistency determined by a Cronbach’s alpha index of 0.79 in the control group and 0.71 in the study group [11,15]. The Sleep Disturbance Scale for Children was constructed and validated in Italy in 1996. The validating study involved two groups of participants. The first group was a healthy control group of students aged 6 to 15 from four public primary schools in Rome. Parents of these students received questionnaires that included the Sleep Disorders Scale for Children (SDSC) and inquiries about medical history, medication use, and any developmental issues. Additionally, a letter explaining the purpose of the study was provided. The second group comprised patients from the Sleep Disorder Center at the Department of Developmental Neurology and Psychiatry at the University of Rome “La Sapienza” [11].

The questions of the SDSC relate to the last six months [8,16,17], with this timeframe effectively distinguishing between transient and chronic disorders [11].

The SDSC is a parental-report tool designed to evaluate sleep patterns in children between the ages of 6 and 15 [8]. Administering the questionnaire is efficient, typically requiring only 10 min [15]. It comprises 26 items evaluated via a Likert-type scale [8,9,15,16]. The initial two items quantify sleep duration (ranging from 1:9–11 h to 5:<5 h) and sleep onset latency (ranging from 1:<15 min to 5:>60 min). The remaining 24 items assess the frequency of specific symptoms using a 5-point frequency scale, where one represents “never” and 5 signifies “always/daily” [16].

Total scores range from a minimum of 26 to a maximum of 130, with higher cumulative values indicating greater severity of sleep pathology [8,17]. Beyond providing a global score, the SDSC categorizes sleep disturbances into six clinical subscales:

- Disorders of initiating and maintaining sleep (DIMS): Items 1, 2, 3, 4, 5, 10, 11.
- Sleep breathing disorders (SBDs): Items 13, 14, 15.
- Disorders of arousal (DA): Items 17, 20, 21.
- Sleep–wake transition disorders (SWTDs): Items 6, 7, 8, 12, 18, 19.
- Disorders of excessive somnolence (DOES): Items 22, 23, 24, 25, 26.
- Sleep hyperhidrosis (SHY): Items 9, 16 [16].

These subscales align with the ASDC classification system and are considered more effective for pediatric populations than the ICDS framework [15]. To date, this versatile screening instrument has been cross-culturally validated in numerous languages, including Italian, Turkish, Chinese, and Spanish, among others [15,16,17,18,19,20,21].

The scale validation process involves both translating the instrument and assessing its psychometric properties in the new language. A critical aspect of this validation is cultural adaptation, which includes the practical application of the scale in Poland and the cross-cultural comparison of the results. The following criteria are important in this process: accurate translation into Polish, functional equivalence, facade equivalence, and precise reconstruction [22].

In the validation study into Polish, the stage of translating the scale was omitted because, after contacting Professor Oliviero Bruni (one of the authors) to obtain consent, we received the certified Polish translation of the SDSC. The Polish language version was prepared in 2022 by ICON Language Services for use in ongoing clinical trials. The translation adhered to the principles of linguistic validation, aiming to create a version that is conceptually equivalent to the original, comparable across languages, culturally relevant to the target country’s context, and easily understood by the intended audience.

The translation process included the following stages:

1. Forward Translation: Two forward translations were conducted by qualified translators, followed by a reconciliation process.
2. Back Translation: One back translation was performed by a qualified translator.
3. Cognitive Interview: Interviews were conducted with five parents of children/adolescents from the general population, aged 5 to 17 years.
4. Proofreading step.

All stages were conducted in accordance with the recommendations of linguistic validation [23].

The next phase of scale validation involved a pilot study of the Polish version. This study included a group of 42 parents of 10-year-olds, and it achieved excellent internal consistency, indicated by a Cronbach’s α value of 0.89, with acceptable values for the individual subscales ranging from 0.69 to 0.83 [24].

The accurate validation of the scale on a larger population in Poland began after receiving approval from the Bioethics Committee of the Poznań University of Medical Sciences.

The study was conducted between May and July 2024 in paper format (not electronically), using the same graphic design and question structure as the original to ensure functional and facade equivalence and faithful reconstruction. It targeted the same age group and occurred in schools in various cities and suburban areas. The research was conducted among parents of children aged 6 to 15 from one kindergarten in a smaller city and two primary schools within the Poznań agglomeration—one in a city with over 100,000 residents and the other in a smaller town. Additionally, the study included two secondary schools in Poznań: a general secondary school and a technical school. After receiving consent from school authorities, teachers distributed information forms, a sociodemographic survey, and the SDSC in Polish to parents, allowing them to read and complete the materials anonymously.

The sociodemographic section of the survey required parents to disclose the child’s age, gender, and their own educational background (categorized as primary, secondary, or higher education). Additionally, respondents provided details regarding their place of residence by population size, in addition to the child’s medical history, including existing conditions and current medications. Data concerning chronic conditions and pharmacological treatment were collected solely through these anonymous parental reports. Furthermore, the questionnaire was employed to explore the child’s sleeping environment—specifically whether they occupied a room or bed alone—and gather information about siblings, including their number and birth order (whether they were younger or older).

### 2.1. Characteristics of the Study Group

A total of 600 questionnaires were distributed, and 389 were returned, yielding a response rate of approximately 65%. Of these, 41 scales were rejected because they contained more than two incomplete responses on the SDSC. Among the remaining 348 participants, 347 provided complete data for all subscales. One additional questionnaire was excluded from the DIMS assessment items because it lacked responses to the first two questions, but it was used to analyze the subscales without the DIMS. This explains the slight variation between N = 348 and N = 347 in specific parts of the statistical reporting.

Sample sizes were determined based on validation recommendations, ensuring a proportional ratio of 5:1 relative to the number of questions on the validated scale. Sources in the literature suggest that a validation sample size of over 300 is considered “good,” while a sample size of over 500 is regarded as “very good” [24].

The participants were divided into the following age groups: 6 years old (29 participants), 7 years old (25 participants), 8 years old (24 participants), 9 years old (30 participants), 10 years old (49 participants), 11 years old (36 participants), 12 years old (34 participants), 13 years old (26 participants), 14 years old (40 participants), and 15 years old (50 participants). Among the surveyed children, there were 183 girls and 165 boys. Most surveys were completed by mothers (284) compared to fathers (48). Most children had siblings (269) compared to those with none (79). The largest group of participants (164) resided in cities with populations exceeding 100,000, followed by 97 participants from towns with populations between 1000 and 20,000 inhabitants. A minimum score of 27 and a maximum of 124 were recorded across the group.

### 2.2. Statistical Analysis

Calculations for this study were conducted using Statistica 13 by TIBCO and PQStat v.1.8.6.122 by PQStat Software (2024). A significance level of α = 0.05 was established, with results considered statistically significant when *p* < α. The reliability of the questionnaire was assessed by calculating Cronbach’s alpha coefficient for the entire questionnaire and its domains.

The normality of variable distributions was evaluated using the Shapiro–Wilk test; however, normality was not achieved for most ordinal variables. Correlations between individual domains were analyzed utilizing Spearman’s rank correlation coefficient (Rs). The Mann–Whitney or Kruskal–Wallis tests, accompanied by the Dunn–Bonferroni multiple comparison tests and the chi-square tests, were employed to compare individual domains across groups derived from sociometric data.

Confirmatory factor analysis (CFA) was conducted to examine the factorial validity of the Sleep Disturbance Scale for Children (SDSC). Analyses were performed in the R statistical environment using a cloud-based RStudio platform (version 4.5.2, Posit Cloud) with the lavaan package (version 0.6-20).

Given the ordinal nature of SDSC items (5-point Likert scale), the model was estimated using the Diagonally Weighted Least Squares estimator with mean and variance adjustment (DWLS/WLSMV), which is recommended for categorical data.

A six-factor model, consistent with the original SDSC structure, was specified. Model fit was evaluated using multiple goodness-of-fit indices: the Comparative Fit Index (CFI) and Tucker–Lewis Index (TLI), with values ≥ 0.90 indicating acceptable and ≥ 0.95 excellent fit; the Root Mean Square Error of Approximation (RMSEA), with values ≤ 0.08 considered acceptable; and the Standardized Root Mean Square Residual (SRMR), with values ≤ 0.08 indicating good fit.

Standardized factor loadings (λ) were inspected, with values ≥ 0.04 considered acceptable.

To determine T-score values, we followed the standardization method used in the original SDSC study, transforming raw scores into T-scores using the formula: T-score = 50 + (value − mean)/standard deviation × 10. A T-score > 70 (representing values above the 97.7th percentile) was established as the reference criterion for a clinically significant sleep disorder. In the absence of an external clinical gold standard (e.g., polysomnography), the T-score > 70 threshold served as the ‘state variable’ for the ROC analysis. Consequently, the Receiver Operating Characteristic (ROC) curve analysis was employed as a technical calibration procedure to identify the raw score equivalents of this statistical threshold. The optimal raw score cut-off points were determined using the Youden index, maximizing the balance between sensitivity and specificity relative to the predefined T-score criterion.

## 3. Results

Table 1 presents the results of the internal consistency of the SDSC and individual subscales. The analysis yielded an excellent internal consistency index for the entire scale, with a Cronbach’s α value of 0.9, which was also acceptable for the individual subscales, ranging from 0.69 for SBDs to 0.83 for SHY.

Spearman’s correlations were performed on the total and subscale scores (Table 2). All subscales correlated positively and significantly with the total score (range = 0.41–0.79), indicating that the Polish SDSC demonstrated convergent validity. Additionally, medium-to-weak (range = 0.18–0.50) positive correlations were identified among all domains. The strongest correlations were found between DIMS and DOES, DIMS and SWTDs, SBDs and SHY, SWTDs and SHY, and SWTDs and DOES.

The structural validity of the Sleep Disturbance Scale for Children (SDSC) was tested using the original 6-factor model [11]. Table 3 presents the Standardized Factor Loadings (λ), which indicate the strength of the relationship between each item and its respective latent factor. In psychometric research, loadings > 0.40 are considered acceptable, with loadings > 0.70 indicating excellent indicator validity.

The six-factor CFA model demonstrated good overall fit to the data (CFI = 0.976, TLI = 0.972, RMSEA = 0.064), indicating satisfactory model adequacy. The SRMR value (0.086) was slightly above the conventional cut-off but was considered acceptable given the complexity of the model and the ordinal nature of the item responses.

All factor loadings were statistically significant (*p* < 0.001). Most standardized loadings were moderate to high (approximately 0.46–0.99), supporting the hypothesized latent structure of the SDSC. The lowest standardized loading was observed for item 1 (sleep duration) within the DIMS factor (λ = 0.29), whereas the remaining items within this factor demonstrated substantial loadings. This finding may reflect the multifactorial nature of sleep duration, which is influenced not only by sleep initiation and maintenance difficulties but also by contextual factors such as family routines, school schedules, and parental regulation, potentially reducing its specificity as an indicator of insomnia-related symptoms.

Patients were divided into subgroups based on the results of the sociometric survey. The correlation of subscale scores with age was examined using Spearman’s rank correlation coefficient (Rs), and the following relationship was demonstrated: the older the patient, the higher the DOES, DIMS, and total scale scores, and the lower the SHY score.

No significant differences were found between the place of residence and the subscale or total scale scores, as determined using the Kruskal–Wallis test. However, the Mann–Whitney test results revealed that patients with siblings scored higher on the SWTDs and SHY.

There were no differences in the median scores between girls and boys for any subscale or total score.

The results of the subscales were analyzed for the presence of comorbidities, including epilepsy, diabetes, depression, headaches, tics, and ADHD. Information on chronic conditions reported by parents in the study group is presented in Table 4. The Mann–Whitney test results indicated significant differences in the median values between individuals with any of these comorbidities and the total Sleep Disturbance Scale for Children (SDSC) score (*p* = 0.000011). Additionally, significant differences were observed in the median values of the following subscales: DIMS (*p* = 0.000004), SBDs (*p* = 0.014), SWTDs (*p* = 0.005), and DOES (*p* = 0.001). These findings are consistent with previous literature suggesting a higher prevalence of sleep disturbances in children with conditions such as epilepsy, diabetes, depression, headaches, tics, or ADHD [25,26,27,28,29,30,31]. However, it should be noted that these diagnoses were based solely on parental reports and were not independently verified through clinical records; thus, the potential for misclassification cannot be entirely excluded.

The mean (SD, range) raw scores for the SDSC and its subscales were as follows: total score: 40.75 (11.70, 27–124); DIMS: 13.09 (4.31, 5–32); SBDs: 3.80 (1.50, 3–15); DA: 3.83 (1.52, 3–15); SWTDs: 8.87 (3.24, 6–30); DOES: 8.38 (3.30, 5–25); and SHY: 2.79 (1.43, 2–10). The minimum value was 27, and the maximum value was 124.

The total and subscale scores reached the following thresholds: 65 for the total, 22 for DIMS, 7 for SBDs, 7 for DA, 16 for SWTDs, 15 for DOES, and 6 for SHY (detailed data in Appendix A). Based on these cut-offs, a total of 65 participants, representing 18.68%, were identified as having at least one sleep disorder: the DIMS rate was 5.46%, the SBD rate was 4.89%, the DA rate was 5.17%, the SWTD rate was 5.75%, the DOES rate was 7.18%, and the SHY rate was 7.47%. Of all the patients examined, 15 reached the cut-off point of a T-score greater than 70 (65), which equates to 4.31% of patients.

Considering the cut-off point established in the original study (39) [11], an alarming 43% (149) of patients would have been diagnosed with sleep disorders. Some authors [16] state that a cut-off point determined by a T-score greater than 70 is considered more stringent. If a more reasonable threshold of a T-score greater than 60 (one standard deviation above the mean) had been adopted in the Polish validation, we would have uncovered sleep disorders in 42 patients, representing 12.07% of the population.

Pathological values of DIMS, DA, and DOES were more prevalent among youth from secondary schools (including high schools and technical schools) compared to children from kindergartens and primary schools. Specifically, the prevalence rates were as follows: DIMS at 17.57% for secondary school students versus 1.83% for younger children; DA at 13.51% for secondary school students compared to 2.56% for younger children; and DOES at 17.57% for secondary school students versus 4.03% for younger children. Values above the cut-off points in the SHY subscale were not statistically more common among boys or primary school students.

## 4. Discussion

The primary objective of this study was to develop a Polish language tool for screening sleep disorders in children by validating and examining the reliability of the Sleep Disturbance Scale for Children (SDSC). Our findings confirm that the Polish version of the SDSC is an effective instrument for this assessment, consistent with evidence gathered from other language versions.

The authors of a study conducted on the Polish population obtained an excellent Cronbach’s alpha for the entire Sleep Disturbance Scale for Children (SDSC), which was 0.897. The individual subscales exhibited Cronbach’s alpha values ranging from 0.69 to 0.83, with the lowest found for the SBD subscale and the highest for the SHY subscale.

In comparison, the authors of the original study reported a Cronbach’s alpha of 0.79 in the control group and 0.71 in the sleep disorder group [11]. Other studies reported values of 0.839 in Turkey [19], 0.85 in France [15], and 0.82 in Spain [16]. Compared to previous studies, none of the subscales in our research showed significantly lower Cronbach’s alpha values; the lowest value for SBDs was still acceptable at 0.69. In contrast, the Turkish study reported a notably low Cronbach’s alpha of 0.467 for DA [19]; similarly low Cronbach’s alpha values for DA were found in the Spanish (0.39) [16] and Iranian (0.4) [8] studies.

Furthermore, our study results demonstrated strong convergent validity similar to the primary version [11] and other language translations [16,19]. All subscales showed significant positive correlations with the total scale score, ranging from 0.41 to 0.79. Notably, the DIMS, DOES, and SWTD subscales showed the highest correlations with the total scale score, consistent with findings from the original study and studies in Spanish [16], Turkish [19], and Chinese [20].

Compared to studies conducted in other languages, such as Turkish [19] and Spanish [16], a slightly higher correlation was observed between the subscales (ranging from 0.18 to 0.5) in the Polish population. Consistent with findings from previous validations [11,16,19], the strongest correlation was observed between the DIMS and DOES subscales, with a correlation coefficient of 0.5, aligning with the observation that individuals with difficulty falling asleep are more likely to experience daytime sleepiness. Correlations among the subscales support the hypothesis mentioned in the original paper that various sleep disorders in children tend to form overlapping subgroups [11].

The findings support the six-factor structure of the SDSC, indicating good overall construct validity, while also revealing localized weaknesses at the item level. Model fit indices met or exceeded commonly accepted criteria for CFA with ordinal data and were consistent with previous validation studies.

The relatively low factor loading of item 1 (sleep duration, λ = 0.29), also observed in Spanish (λ = 0.3) [16] and Chinese (λ = 0.37) [20] studies, may reflect the fact that sleep duration is influenced by multiple external and developmental factors (e.g., age-related sleep needs, school schedules, parental regulation) that extend beyond difficulties with sleep initiation and maintenance. Consequently, sleep duration may represent a more heterogeneous indicator compared to items directly assessing nocturnal sleep problems, which could explain its weaker association with the DIMS factor.

Despite this finding, the item was retained due to its theoretical relevance and its established role in the original SDSC structure. Overall, the results support the use of the SDSC as a multidimensional measure of sleep disturbances in children.

In line with the original study’s findings, the prevalence of DOES increases with age in the Polish population, whereas the prevalence of SHY decreases with age. The rise in daytime sleepiness with age is linked to a natural increase in daytime sleepiness during adolescence. In addition, the decline in SHY observed in the Italian study is posited to be related to the maturation of the autonomic nervous system [11].

In our study, we observed higher values on the SDSC for chronic conditions, consistent with the findings of the Turkish study [19].

Our sociometric survey extended beyond previous studies by incorporating a question regarding the presence of siblings among participants. The findings revealed that individuals with siblings exhibited higher scores on the SWTD and SHY subscales. The SWTD subscale specifically addresses various sleep-related phenomena, including hypnic jerks, rhythmic movement disorders, sleep talking, bruxism, nocturnal hyperkinesia, and hypnagogic hallucinations. The presence of another individual in the bedroom allows for more accurate reporting of these occurrences during sleep, similar to the documentation of excessive sweating in the SHY subscale.

The results obtained from the Polish population indicate that at least 18.68% of the participants in the study had at least one abnormal subscale level on the Sleep Disturbance Scale for Children (SDSC). In our study, a cut-off of 39—consistent with the original study—was exceeded by 160 individuals, representing 46% of the participants. These findings suggest that sleep disorders affect a significantly larger percentage of the population than previously estimated. However, our results align more closely with the existing literature, in which the prevalence of sleep disorders among children and adolescents is estimated to be between 25% and 50% [16]. Our cut-off point, calculated based on a t-score greater than 70, reached a higher value (65) and was achieved by 15 patients. In studies conducted in the last 5 years, a higher cut-off point was also observed: 53 in the Spanish study [16] and 55 in the Turkish study [19].

The high rates observed may be attributed to several factors. First, the Polish study was conducted after the COVID-19 pandemic, which significantly influenced the rise in sleep disorder incidences [2,32,33]. Second, the time elapsed since the original study—from 1996 to 2024—has been marked by an increase in access to screens among children and adolescents. According to the available literature, this increase in screen time is also linked to the rising prevalence of sleep disorders [3,4,5].

In Poland, similar to the Spanish study [16], pathological values of DIMS, DOES, and DA were more frequent in older groups, specifically among secondary school students in our study; in contrast, however, we did not find a higher incidence of disorders in the SHY subscale in boys and primary school students.

However, certain limitations of the Polish study should be noted. First, only complete forms were included in the full analysis. This may lead to response bias, as parents of children with sleep problems are more likely to complete the sleep survey. Unlike the original study, our research did not include a retest.

Another limitation is the inability to assess true diagnostic performance, including discriminant validity, sensitivity, and specificity relative to an external gold standard, as the results were examined in only one group without comparison to objective sleep studies, such as polysomnography or actigraphy. Consequently, the sensitivity and specificity values reported here reflect classification agreement relative to the statistical cut-off (T-score > 70) rather than clinical validity against an external criterion. Therefore, utilizing a T-score threshold above 70 may lead to a higher rate of false negatives in clinical settings and prevalence estimates should not be considered epidemiological data. It is important to note that the established cut-off values are based on research conducted in the general population and have not been verified in clinical groups. As a result, the current estimates of sensitivity and specificity should be regarded as preliminary measures of technical calibration.

Additional limitations of our study stem from the questionnaire format, which may lead parents to inaccurately recall their child’s symptoms, heightening the risk of social desirability and recall bias.

## 5. Conclusions and Future Directions

Despite its limitations, this Polish study demonstrated satisfactory psychometric properties of the Polish version of the Sleep Disorders Scale for Children (SDSC). The six-factor structure showed good overall model fit, with most items displaying moderate to high standardized factor loadings. One exception was Item 1 (sleep duration), which showed a relatively weaker loading, possibly reflecting developmental or contextual variability in parental perceptions of children’s sleep duration. Overall, the findings support the Polish version of the SDSC as a reliable and valid screening tool for sleep disturbances in children aged 6–15 years, consistent with previous international validation studies.

The SDSC enables the initial screening of the six most common sleep problem groups and the selection of cases for targeted diagnosis. Creating such a tool in Polish is essential due to the negative consequences of poor sleep in childhood and adolescence, which are becoming increasingly prevalent with the widespread use of screens.

Future studies using objective sleep assessment methods should be conducted to determine the accuracy of clinically significant results. Regarding clinical application, our findings suggest that a Total Score ≥ 65 (corresponding to T-score > 70) may serve as a preliminary signal for further diagnostic evaluation. Furthermore, for scores in the borderline range (above 39 but below 65), a cautious approach involving periodic reassessment after 8–12 weeks is recommended, and if an upward trend is observed, diagnostics should also be extended.

In the future, it would also be of great benefit to conduct studies using the Polish scale in various pediatric conditions, rather than only in potentially healthy populations.

## Figures and Tables

**Table 1:** Mean, standard deviation, and Cronbach’s α value for the entire scale and individual subscales.

	DIMS	SBDs	DA	SWTDs	SHY	DOES	Total
Group size	347	348	348	348	348	348	347
Number of items	7	3	3	6	5	2	26
Mean	13.11	3.80	3.83	8.87	2.79	8.38	40.798
Standard deviation (SD) of the scale	4.30	1.50	1.53	3.24	1.43	3.30	11.71
Cronbach’s α value	0.74	0.69	0.76	0.72	0.83	0.76	0.897
−95% CI Cronbach’s	0.70	0.63	0.71	0.67	0.79	0.72	0.88
+95% CI Cronbach’s	0.78	0.74	0.80	0.76	0.86	0.796	0.91
Standard error (SE) of measurement	2.18	0.84	0.75	1.73	0.58	1.62	3.76
Average correlation	0.31	0.50	0.52	0.31	0.72	0.41	0.28
Cronbach’s alpha standardized (STD)	0.76	0.75	0.77	0.73	0.84	0.77	0.81

DIMS: disorders of initiating and maintaining sleep, SBDs: sleep breathing disorders, DA: disorders of arousal, SWTDs: sleep–wake transition disorders, SHY: sleep hyperhidrosis, DOES: disorders of excessive somnolence.

**Table 2:** Matrix plot of correlations (Spearman Rs).

	DIMS	SBDs	DA	SWTDs	DOES	SHY	Total
DIMS	1.00						
SBDs	0.18	1.00					
DA	0.34	0.21	1.00				
SWTDs	0.40	0.35	0.43	1.00			
DOES	0.50	0.26	0.23	0.43	1.00		
SHY	0.18	0.43	0.33	0.45	0.22	1.00	
Total	0.79	0.41	0.49	0.74	0.73	0.49	1.00

**Table 3:** Standardized Factor Loadings of the Sleep Disturbance Scale for Children.

Factor (Subscale)	Item	Loading (λ)
DIMS (Disorders of initiating and maintaining sleep)	1. Sleep duration	0.29
	2. Sleep latency	0.49
	3. Going to bed reluctantly	0.43
	4. Difficulty falling asleep	0.81
	5. Falling asleep anxiety	0.80
	10. Night awakenings	0.76
	11. Difficulty in falling asleep after awakenings	0.75
SBDs (Sleep breathing disorders)	13. Breathing problems	0.93
	14. Sleep apnea	0.99
	15. Snoring	0.53
DA (Disorders of arousal)	17. Sleepwalking	0.79
	20. Sleep terrors	0.89
	21. Nightmares	0.76
SWTDs (Sleep–wake transition disorders)	6. Hypnic jerks	0.78
	7. Rhythmic movement disorders	0.98
	8. Hypnagogic hallucinations	0.65
	12. Nocturnal hyperkinesia	0.63
	18. Sleep talking	0.46
	19. Bruxism (Teeth grinding)	0.49
DOES (Disorders of excessive somnolence)	22. Difficulty in waking up	0.60
	23. Tired when waking up	0.71
	24. Sleep paralysis	0.85
	25. Daytime somnolence	0.68
	26. Sleep attacks	0.87
SHY (Sleep hyperhidrosis)	9. Falling asleep sweating	0.94
	16. Night sweating	0.89

**Table 4:** Chronic conditions reported by parents in the study group.

Disease Reported	Number of Patients
No chronic conditions	248
ADHD	22
Headaches	20
Allergies	9
Tics	3
Diabetes	2
Depression	2
Epilepsy	1

## Data Availability

The original contributions presented in this study are included in the article. Further inquiries can be directed to the corresponding author.

## Abbreviations

The following abbreviations are used in this manuscript:

SDSC	Sleep Disturbance Scale for Children
DIMS	disorders of initiating and maintaining sleep
SBDs	sleep breathing disorders
SWTDs	sleep–wake transition disorders
DOES	disorders of excessive somnolence
SHY	sleep hyperhidrosis
DA	disorders of arousal

## Appendix A. T-Score > 70

(a) DIMS

**DeLong’s Method**	
AUC	1
SE(AUC)	0
−95% CI	1
+95% CI	1
Z-score	7.330315
Two-sided *p*-value	<0.000001
**For the Cut-Off Point**	
Cut-off point method	Youden Index
Cut-off point	≥22

For DIMS, the proposed cut-off point is ≥22 points, indicating pathology:

**(****≥****) DIMS**	**Sensitivity**	**Specificity**	**PPV**	**NPV**
5	1	0	0.0545977011494253	NA
7	1	0.00303951367781155	0.0547550432276657	1
8	1	0.0182370820668693	0.0555555555555556	1
9	1	0.0790273556231003	0.0590062111801242	1
10	1	0.173252279635258	0.0652920962199313	1
11	1	0.319148936170213	0.0781893004115226	1
12	1	0.462006079027356	0.0969387755102041	1
13	1	0.595744680851064	0.125	1
14	1	0.696048632218845	0.159663865546218	1
15	1	0.778115501519757	0.206521739130435	1
16	1	0.835866261398176	0.26027397260274	1
17	1	0.86322188449848	0.296875	1
18	1	0.90273556231003	0.372549019607843	1
19	1	0.924012158054711	0.431818181818182	1
20	1	0.96048632218845	0.59375	1
21	1	0.981762917933131	0.76	1
22	1	1	1	1
23	0.894736842105263	1	1	0.993957703927493
24	0.631578947368421	1	1	0.979166666666667
25	0.473684210526316	1	1	0.970501474926254
26	0.315789473684211	1	1	0.961988304093567
29	0.157894736842105	1	1	0.953623188405797
32	0.0526315789473684	1	1	0.948126801152738

(b) SBDs

**DeLong’s Method**	
AUC	1
SE(AUC)	0
−95% CI	1
+95% CI	1
Z-score	6.954828
Two-sided *p*-value	<0.000001
**For the Cut-Off Point**	
Cut-off point method	Youden Index
Cut-off point	≥7

For SBDs, the proposed cut-off point is ≥7 points, indicating pathology:

**(****≥****) SBDs**	**Sensitivity**	**Specificity**	**PPV**	**NPV**
3	1	0	0.0488505747126437	NA
4	1	0.610271903323263	0.116438356164384	1
5	1	0.885196374622356	0.309090909090909	1
6	1	0.972809667673716	0.653846153846154	1
7	1	1	1	1
8	0.764705882352941	1	1	0.988059701492537
9	0.529411764705882	1	1	0.976401179941003
10	0.176470588235294	1	1	0.959420289855072
15	0.117647058823529	1	1	0.956647398843931

(c) DA

**DeLong’s Method**	
AUC	1
SE(AUC)	0
−95% CI	1
+95% CI	1
Z-score	7.14564
Two-sided *p*-value	<0.000001
**For the Cut-Off Point**	
Cut-off point method	Youden Index
Cut-off point	≥7

For DA, the proposed cut-off point is ≥7 points, indicating pathology:

**(****≥****) DA**	**Sensitivity**	**Specificity**	**PPV**	**NPV**
3	1	0	0.0517241379310345	NA
4	1	0.624242424242424	0.126760563380282	1
5	1	0.863636363636364	0.285714285714286	1
6	1	0.954545454545455	0.545454545454545	1
7	1	1	1	1
8	0.611111111111111	1	1	0.979228486646884
9	0.444444444444444	1	1	0.970588235294118
10	0.277777777777778	1	1	0.962099125364432
11	0.222222222222222	1	1	0.959302325581395
13	0.111111111111111	1	1	0.953757225433526
15	0.0555555555555556	1	1	0.951008645533141

(d) SWTDs

**DeLong’s Method**	
AUC	1
SE(AUC)	0
−95% CI	1
+95% CI	1
Z-score	7.509307
Two-sided *p*-value	<0.000001
**For the Cut-Off Point**	
Cut-off point method	Youden Index
Cut-off point	≥16

For SWTDs, the proposed cut-off point is ≥16 points, indicating pathology:

**(≥) SWTDs**	**Sensitivity**	**Specificity**	**PPV**	**NPV**
6	1	0	0.0574712643678161	NA
7	1	0.225609756097561	0.072992700729927	1
8	1	0.457317073170732	0.101010101010101	1
9	1	0.63719512195122	0.143884892086331	1
10	1	0.76219512195122	0.204081632653061	1
11	1	0.835365853658537	0.27027027027027	1
12	1	0.896341463414634	0.37037037037037	1
13	1	0.942073170731707	0.512820512820513	1
14	1	0.966463414634146	0.645161290322581	1
15	1	0.981707317073171	0.769230769230769	1
16	1	1	1	1
17	0.7	1	1	0.982035928143713
18	0.55	1	1	0.973293768545994
19	0.3	1	1	0.95906432748538
20	0.25	1	1	0.956268221574344
30	0.05	1	1	0.945244956772334

(e) DOES

**DeLong’s Method**	
AUC	1
SE (AUC)	0
−95% CI	1
+95% CI	1
Z-score	8.331423
Two-sided *p*-value	<0.000001
**For the Cut-Off Point**	
Cut-off point method	Youden Index
Cut-off point	≥15

For DOES, the proposed cut-off point is ≥15 points, indicating pathology:

**(****≥****) DOES**	**Sensitivity**	**Specificity**	**PPV**	**NPV**
5	1	0	0.0718390804597701	NA
6	1	0.191950464396285	0.0874125874125874	1
7	1	0.34984520123839	0.106382978723404	1
8	1	0.51702786377709	0.138121546961326	1
9	1	0.687306501547988	0.198412698412698	1
10	1	0.804953560371517	0.284090909090909	1
11	1	0.86687306501548	0.367647058823529	1
12	1	0.91640866873065	0.480769230769231	1
13	1	0.95046439628483	0.609756097560976	1
14	1	0.984520123839009	0.833333333333333	1
15	1	1	1	1
16	0.36	1	1	0.952802359882006
17	0.32	1	1	0.95
19	0.28	1	1	0.947214076246334
20	0.16	1	1	0.938953488372093
24	0.08	1	1	0.933526011560694
25	0.04	1	1	0.930835734870317

(f) SHY

**DeLong’s Method**	
AUC	1
SE(AUC)	0
−95% CI	1
+95% CI	1
Z-score	8.483255
Two-sided *p*-value	<0.000001
**For the Cut-Off Point**	
Cut-off point method	Youden Index
Cut-off point	≥6

For SHY, the proposed cut-off point is ≥6 points, indicating pathology:

**(****≥****) SHY**	**Sensitivity**	**Specificity**	**PPV**	**NPV**
2	1	0	0.0747126436781609	NA
3	1	0.720496894409938	0.224137931034483	1
4	1	0.850931677018634	0.351351351351351	1
5	1	0.956521739130435	0.65	1
6	1	1	1	1
7	0.307692307692308	1	1	0.947058823529412
8	0.192307692307692	1	1	0.938775510204082
10	0.115384615384615	1	1	0.933333333333333

(g) Total SDSC

**DeLong’s Method**	
AUC	1
SE(AUC)	0
−95% CI	1
+95% CI	1
Z-score	6.55263
Two-sided *p*-value	<0.000001
**For the Cut-Off Point**	
Cut-off point method	Youden Index
Cut-off point	≥65

For total SDSC, the proposed cut-off point is ≥65 points, indicating pathology:

**(****≥****) SDSC**	**Sensitivity**	**Specificity**	**PPV**	**NPV**
27	1	0	0.0431034482758621	NA
28	1	0.003003003003003	0.0432276657060519	1
29	1	0.027027027027027	0.0442477876106195	1
30	1	0.0690690690690691	0.0461538461538462	1
31	1	0.108108108108108	0.0480769230769231	1
32	1	0.177177177177177	0.0519031141868512	1
33	1	0.234234234234234	0.0555555555555556	1
34	1	0.273273273273273	0.0583657587548638	1
35	1	0.345345345345345	0.0643776824034335	1
36	1	0.42042042042042	0.0721153846153846	1
37	1	0.48948948948949	0.0810810810810811	1
38	1	0.528528528528529	0.0872093023255814	1
39	1	0.564564564564565	0.09375	1
40	1	0.597597597597598	0.100671140939597	1
41	1	0.630630630630631	0.108695652173913	1
42	1	0.651651651651652	0.114503816793893	1
43	1	0.6996996996997	0.130434782608696	1
44	1	0.735735735735736	0.145631067961165	1
45	1	0.774774774774775	0.166666666666667	1
46	1	0.807807807807808	0.189873417721519	1
47	1	0.828828828828829	0.208333333333333	1
48	1	0.855855855855856	0.238095238095238	1
49	1	0.867867867867868	0.254237288135593	1
50	1	0.873873873873874	0.263157894736842	1
51	1	0.885885885885886	0.283018867924528	1
52	1	0.894894894894895	0.3	1
53	1	0.918918918918919	0.357142857142857	1
54	1	0.936936936936937	0.416666666666667	1
55	1	0.945945945945946	0.454545454545455	1
56	1	0.957957957957958	0.517241379310345	1
57	1	0.960960960960961	0.535714285714286	1
58	1	0.96996996996997	0.6	1
59	1	0.975975975975976	0.652173913043478	1
60	1	0.984984984984985	0.75	1
62	1	0.990990990990991	0.833333333333333	1
65	1	1	1	1
66	0.933333333333333	1	1	0.997005988023952
68	0.866666666666667	1	1	0.994029850746269
69	0.8	1	1	0.991071428571429
72	0.733333333333333	1	1	0.98813056379822
73	0.666666666666667	1	1	0.985207100591716
74	0.6	1	1	0.982300884955752
75	0.533333333333333	1	1	0.979411764705882
76	0.466666666666667	1	1	0.976539589442815
79	0.4	1	1	0.973684210526316
83	0.333333333333333	1	1	0.970845481049563
86	0.266666666666667	1	1	0.968023255813954
90	0.2	1	1	0.965217391304348
92	0.133333333333333	1	1	0.96242774566474
124	0.0666666666666667	1	1	0.959654178674352
